# Supplementary material for: Understanding how and why audits work in improving the quality of hospital care: A systematic realist review
Source: PLoS One. 2021 Mar 31;16(3):e0248677. doi: 10.1371/journal.pone.0248677 (PMC8011742; doi:10.1371/journal.pone.0248677)
Supplement: S1 Box — (DOCX) [file pone.0248677.s002.docx]

**S1 Box. Search strategy**

| **Search strategy** |
| --- |
| MEDLINE (Pubmed)  ("Clinical Audit"[Majr:noexp] OR "Medical Audit"[Majr] OR "Nursing Audit"[Majr] OR "Accreditation"[Majr] OR "Certification"[Majr:noexp] OR "Peer Review, Health Care"[Majr] OR ((extern*[tiab] OR internal*[tiab]) AND audit[tiab]) OR medical audit*[tiab] OR clinical audit*[tiab] OR nursing audit*[tiab] OR audit[ti] OR audits[ti] OR accreditat*[ti] OR visitation*[ti]) AND  ("Academic Medical Centers"[Mesh:noexp] OR "Hospitals, Teaching"[Mesh] OR "Outpatient Clinics, Hospital"[Mesh:noexp] OR "Hospitals"[Mesh:noexp] OR hospital*[tiab] OR ((health*[tiab] OR clinical[tiab]) AND (organisation*[tiab] OR organization*[tiab] OR center*[tiab] OR centre*[tiab])) OR health sector*[tiab] OR healthcare sector*[tiab] OR health care sector*[tiab]) AND  ("Efficiency, Organizational"[Mesh] OR efficien*[tiab] OR effectiveness*[tiab] OR performan*[tiab] OR improvement*[tiab] OR "Quality Improvement"[Mesh:noexp] OR "Quality Assurance, Health Care"[Majr:noexp] OR quality improv*[ti] OR quality assur*[ti]) NOT ("Animals"[Mesh] NOT "Humans"[Mesh]) AND english[la] AND ("last 10 years"[PDat]) |
