# Supplementary material for: Understanding how and why audits work in improving the quality of hospital care: A systematic realist review
Source: PLoS One. 2021 Mar 31;16(3):e0248677. doi: 10.1371/journal.pone.0248677 (PMC8011742; doi:10.1371/journal.pone.0248677)
Supplement: S1 Table — (DOCX) [file pone.0248677.s003.docx]

**S1 Table. Characteristics of the studies included in the review**

| **Author, year**  **(country)** | **Type of document** | **Type of audit** | **Aim** | **Contributed to CMOc** | **Profession of recipients** | **Outcomes** | **Usefulness and relevance of the study** | **Quality**  **score^a^** | **GRADE score^b^** |
| --- | --- | --- | --- | --- | --- | --- | --- | --- | --- |
| Olliveri, 2015  (Belgium) | Cohort study | Clinical audit | To determine whether an audit could improve and sustain compliance with CT procedures. | CMOc2 | Physicians | Compliance with CT procedures was improved (although compliance was still not perfect) and sustained over a 1-year period. QI committee was established. | Low | 15 | C1 |
| Nardini, 2014  (Italy) | Cohort study | Clinical audit | To measure whether health professionals conform to guidelines. | CMOc2 | Physicians | Improvements in reporting, knowledge and in the use of therapy and diagnostics. No improvements in prescriptions and rehabilitation. | Low | 13 | C3 |
| Kurmis, 2015  (Australia, New Zealand) | Cohort study | Clinical audit | To identify areas for improvement in nutritional support and to evaluate the usability and clinical relevance of the audit criteria. | CMOc6 | Physicians and nurses | Much key areas for clinical improvement, mostly due to poor staffing levels. | Moderate | 8 | C2 |
| Reznek, 2014 (USA) | Controlled trial | Peer review | To evaluate the effectiveness of a peer review process in promoting incident reporting. | CMOc2  CMOc3  CMOc4 | Physicians | Frequency of reporting increased. Physicians perceived the peer review process to be educational and highly effective in identifying errors. It led to an improved understanding of system barriers to quality and improved commitment to quality. | High | 13 | B1 |
| Johnson, 2014 (USA) | Multiple case study | Certification | To compare compliance with the Joint Commissions 10 performance measure data for certified hospitals, hospitals preparing for certification and non-certified hospitals. | CMOc1 | Physicians, nurses, others | Defect-free care was delivered best at hospitals preparing for certification, followed by Primary Stroke Centres and non- Primary Stroke centres. Primary Stroke centres and hospitals preparing for certification had a higher average annual percent increase in the provision of defect-free care compared with non- Primary Stroke centres. | Low | 11 | C1 |
| Johnson, 2018 (USA) | Single case study | Clinical audit | To improve hospital-staff adherence to infection control guidelines. | CMOc2 | Physicians, nurses, others | Audits of staff adherence conducted pre and post intervention demonstrated an increased and sustainable improvement among each group. | Low | 8 | C3 |
| Perkins, 2014 (USA) | Retrospective cohort study | Clinical audit | To evaluate operating room efficiency and utilization and to identify areas for improvement. | CMOc2  CMOc3  CMOc4  CMOc6 | Physicians, nurses, others | The operating room times were comparable to the corresponding times from previous studies. One period was longer at the study site and contributed disproportionately to OR inefficiency and poor utilization of OR time. A multidisciplinary stakeholder team was established to address these issues. | High | 9 | C3 |
| Mills, 2014 (UK) | Cohort study | Clinical audit | To ensure that eligible patients were screened for dementia. | CMOc2 | Physicians | Within the first audit, 52% of the patients were not assessed for dementia. The second audit showed improvement in the number of patients screened for dementia. | Low | 11 | C3 |
| Hall, 2014 (UK) | Cohort study | Clinical audit | To improve the quality of service provided and the overall patient experience. | CMOc2  CMOc4  CMOc6 | Physicians | Lack of documented nasal examination and total absence of nasal cautery and high re-bleed rate. Re-audits showed trend for improvement, more clarity in decision making amongst junior doctors and more equipment available. Also, nasal examination was improved, re-bleedings were decreased. | High | 14 | C3 |
| Kennedy, 2013 (Australia) | Cohort study | Clinical audit | To audit and optimise cancer surveillance. | CMOc2 | Physicians and nurses | Substantial and sustained improvements in adherence to cancer surveillance. The use of prescribed tests (according to guidelines) increased significantly. Adequacy of surveillance (complete cycle of the prescribed tests) also improved significantly. | Low | 13 | C3 |
| Iyer, 2012 (USA) | Descriptive study | Peer review/Dutch visitation model | To categorize radiologist peer review comments and evaluate their functions within the context of a quality assurance programme. | CMOc7 | Physicians | In 1 year, 6813 cases were scored. 6.3% were accompanied by comment entries. Also, some comments were entered for informational or educational feedback. Most cases were entered as errors of observation or interpretation. | Moderate | 9 | D2 |
| Kalanithi, 2013 (USA) | Cohort study | Clinical audit | To improve communication between residents and general practitioners. | CMOc2  CMOc3 | Physicians | Two PDSA cycles were conducted that identified two best practices used by high-performing teams: having the senior resident responsible for primary care physicians’ communication and standardizing documentation of communication on the day of admission. One year after the baseline audit, general practitioners were more satisfied with the communication at admission. | High | 14 | C3 |
| Anderson, 2012 (UK) | Descriptive study | Clinical audit | To review the clinical audit programme. | CMOc2 | Unknown | No outcomes described. | Low | 7 | D2 |
| Cosgrove, 2008 (UK) | Cohort study | Clinical audit | To decrease delays in urgent and expedited surgery by using a clinical audit. | CMOc2 | Physicians; nurses and other healthcare professionals | Improvements in emergency theatre utilisation through a decrease in delays in urgent and expedited surgery and a decrease in waiting times. | Low | 12 | C3 |
| Kilsdonk, 2014 (The Netherlands) | Controlled trial | Peer review/Dutch visitation model | To investigate whether the  participation in and extent of the external peer review programme  impacted multidisciplinary  treatment patterns  and survival of colorectal cancer patients. | CMOc1 | Unknown | Patients from intervention hospitals more frequently received adjuvant  chemotherapy. Survival was slightly higher in patients from intervention hospitals but unrelated to the phase of the programme in which the hospital was at the time of diagnosis. | Low | 13 | B1 |
| Esposito, 2013 (Italy) | Cohort study | Clinical audit | To highlight the discrepancies between actual and standard settings and to identify the changes needed to improve the quality of care. | CMOc3  CMOc4  CMOc5 | Physicians | Significant number of hypertensive patients that became normotensive and, in those who remained hypertensive, the average blood pressure decreased. Significant reduction in the use of antihypertensive drugs. | Moderate | 16 | C3 |
| Ingen, 2012 (France) | Cohort study |  | To assess the efficiency of a dermatosurgery department. | CMOc5 | Physicians, nurses, others | Implementation of preoperative consultation, improved leaflets for patients, standardizing of reports, earmarking of funds for materials, and patient satisfaction survey. Training of residents was organized. | Moderate | 14 | C3 |
| Numan, 2012 (The Netherlands) | Cohort study | Clinical audit | To evaluate the effectiveness of implementing a multidisciplinary care path. | CMOc4  CMOc5 | Physicians, nurses, others | Reduction in length of stay, postoperative pain and loss of quality of life. Increase usage of muscle sparing and video-assisted surgery techniques. Higher satisfaction of the team. | Moderate | 13 | C3 |
| Sheena, 2012 (UK) | Cohort study | Clinical audit | To assess compliance with the WHO surgical safety checklist. | CMOc3 | Physicians, nurses, others | Time out was better performed, compliance in pre- and post-operative documentation. Improvements in communication and better documentation of post-operative plans. | Low | 13 | C3 |
| Dupont, 2011 (France) | Multiple case study | Clinical audit | To assess the impact of regular criteria-based audits on the prevalence of severe postpartum haemorrhage. | CMOc3  CMOc4 | Physicians, nurses, others | Significant reduction in prevalence of sever postpartum haemorrhage. Improved quality of care. The audit encouraged collaboration. | Moderate | 15 | C1 |
| Dinescu, 2011 (USA) | Single case study | Clinical audit | To assess whether an audit and feedback intervention would improve the discharge summaries of medicine fellows. | CMOc2 | Physicians | Fellows were more likely to complete discharge summary data. Increased awareness of fellows of discharge information and fellows were motivated to change. | Low | 11 | C3 |
| Langston, 2010 (USA) | Cohort study | Clinical audit | To increase compliance to hand hygiene through non-personal and personal feedback to staff. | CMOc7 | Physicians and nurses | Significant increase in hand hygiene compliance. The audit made staff aware of hand hygiene practices and gave them a sense of empowerment in giving feedback to the other staff (justification to tell others to follow policy). | Moderate | 9 | C3 |
| Gallagher, 2011 (Australia) | Cohort study | Clinical audit | To link blood transfusion protocols to clinical practice guidelines and to increase their accessibility. | CMOc4 | Physicians, nurses, others | Downward trend of rejections. Improved compliance with recording. Correct compatibility of labels and overall better recording. Raised awareness and better and safer transfusion practice. | Low | 12 | C1 |
| Clarke, 2010 (UK) | Single case study | Clinical audit | To improve patient assessment practices, recognition of patient deterioration and communication in the acute ward environment. | CMOc2  CMOc3  CMOc4 | Nurses | Clear discrepancy between the care that was identified on the nursing care plan and the delivered care. Actions were undertaken to improve education programmes and critical discussion regarding clinical practices. | High | 10 | C4 |
| McLiesh, 2009 (Australia) | Before and after study | Clinical audit | To assess current practice and implement changes to match best practices in the management of pain. | CMOc4 | Physicians, nurses, others | Assessment tool was introduced that was especially designed for the patient group involved. Awareness and knowledge improved and staff were enthusiast about the introduction of the assessment tool. The use of the tool increased. | Low | 12 | C3 |
| Gommans, 2008 (New Zealand) | Cohort study | Clinical audit | To improve the quality of written prescriptions. | CMOc2  CMOc7 | Physicians, nurses, others | Serial audits showed progressive improvements in legibility, documentation of dose, medication route, date and patient identification. | Moderate | 13 | C3 |
| Pomey, 2010 (Canada) | Multiple case study | Accreditation | To evaluate how the accreditation process helps introduce organizational changes that enhance the  quality and safety of care. | CMOc1  CMOc5  CMOc6 | Physicians, nurses, others | The accreditation process was effective for accelerating integration and stimulating a spirit of cooperation; increasing social capital by giving staff the opportunity to develop relationships and for fostering links between stakeholders. The motivation of healthcare organisations to introduced accreditation-related changes decreased over time. | High | 8 | C2 |
| Jain, 2008 (UK) | Cohort study | Clinical audit | To assess the management of stable ankle fractures and measure the change in practice after implementation of guidelines. | CMOc4 | Physicians, nurses, others | Better treatment of patients according to guidelines, less appointments and radiographs necessary, thereby reducing the costs. | Low | 11 | C3 |
| Li, 2008 (USA) | Single case study | Clinical audit | To evaluate the rate of adherence to the prophylactic of thrombosis protocol. | CMOc2 | Unknown | Most of the patients received prophylaxis, however, a large proportion of the patients were receiving suboptimal therapy. Low rate of adherence to the protocol. | Low | 7 | C4 |
| Vanoli, 2011 (Italy) | Multiple case study | Peer review | To present the early results of a peer- vs self-evaluation programme. | CMOc2 CMOc5  CMOc6 | Physicians | Self-evaluation was higher than peer-evaluation. The frequency of regular team discussions between physicians and nurses was overestimated, too few briefing meetings being actually carried out. Some degree of defects of regular registration by nurses of patients’ needs, planning of nursing care and evidence of actually performed interventions emerged in both self- and peer-evaluation. | Low | 4 | C2 |
| Ursprung, 2005 (USA) | Single case study | Clinical audit | To determine the feasibility and utility of real time safety auditing during routine clinical work in an intensive care unit. | CMOc2 | Physicians, nurses, others | Many errors were detected. Diverse error types were found including unlabelled medication at the bedside, ID band missing or  in an inappropriate location, inappropriate pulse oximeter alarm setting, and delay in  communication/information transfer that led to a delay in appropriate care. | Low | 7 | C4 |
| Wright, 2014 (Australia) | Cohort study | Clinical audit | To audit in-hospital falls prevention practices, to implement evidence-based best practice recommendations and to increase staff compliance with falls prevention. | CMOc3 | Nurses | Performance emerged as very poor. At the follow up audit, there was an overall improvement in compliance of implementing best practice falls prevention recommendations. At the second follow up audit, overall improvement in compliance of implementing best practice falls prevention recommendations has been maintained for most criteria. | High | 14 | C1 |
| Halpape, 2014 (Canada) | Cohort study | Clinical audit | To optimize the treatment of patients with pneumonia under hospitalist care by focusing on best practice and local antibiogram data. | CMOc2 | Physicians | The adherence rates were 10% (2/21) at baseline and 38% (5/13) in the post-intervention audit, a statistically significant 4-fold increase. | Low | 12 | C3 |
| Benitez, 2012 (Spain) | Cohort study | Clinical audit | To assess the feasibility of a quality care project in palliative sedation. | CMOc2 | Physicians and nurses | Decisions and procedures for establishing palliative sedation were made with high adherence to the clinical protocol. High adherence to the palliative sedation guide. | Low | 12 | C3 |
| Lewis, 2015 (USA) | Cohort study | Clinical audit | To determine whether an  audit and feedback system is an effective means of motivating  surgical quality improvement for high-acuity procedures (HAPs) and low-acuity procedures (LAPs). | CMOc3 | Physicians | No significant differences in the proportions of LAPs and HAPs or in the prevalence of patient comorbidities. The mean length of stay significantly decreased. The incidence of 1 or more negative performance indicators decreased significantly for LAPs  and trended downward for HAPs. | Low | 11 | C3 |
| Munn, 2015 (Australia) | Cohort study | Clinical audit | To improve compliance with best practice in medication administration. | CMOc2 | Nurses | Improved compliance with standards regarding medication administration: Compliance was increased in 6/8 criteria. Increased awareness in evidence-based healthcare, skill development and critical thinking. | Low | 12 | C1 |
| Easterlow, 2010 (UK) | Cohort study | Clinical audit | To reduce the incidence of peripheral intravenous cannulae (PVC) related infections. | CMOc2 | Physicians and nurses | Change in culture, use of better materials for PVC, better educated staff, improved guidelines and care plans. | Low | 15 | C3 |
| Radford, 2007 (UK) | Cohort study | Clinical audit | To assess the recording and quality of drug/clinical hypersentivities within records. | CMOc2 | Physicians and nurses | Overall improvements in both documentation of drug allergies in preoperative clinical notes and the bedside drug charts. | Low | 7 | C3 |
| Cohen, 2005 (USA) | Cohort study | Clinical audit | To assess the impact of a hospital-based patient safety program on rate of adverse drug events. | CMOc3 | Physicians, nurses, others | Significant and lasting reduction in patient harm as measured by adverse drug events. Hospital wide acceptance of the introduced interventions. | Low | 15 | C3 |
| Roberts, 2011 (UK) | RCT | Peer review | To study the impact of peer review on COPD quality measures. | CMOc5 | Physicians, nurses, others | Significant change in service. Improved quality of care, improvements in service delivery and changes within departments that promote and are precursors to quality improvement. Within several units, the profile of the unit was raised, better team morale, strong sense of team philosophy, opportunity to take ownership of a project and develop leadership skills and positive shared experiences with how other units delivers care. | Low | 12 | A2 |
| Hunter, 2014 (Australia) | Cohort study | Clinical audit | To inform health professionals of processes to reduce the incidence and prevalence of pressure injuries. | CMOc2 | Nurses | Prevalence and incidence of pressure injuries were reduced and sustained low. Organisational change was achieved by using an holistic approach and involving several stakeholders. | Low | 12 | C3 |
| Bogh, 2016 (Denmark) | Cohort study | Accreditation | To examine whether performance measures improve more in accredited hospitals than in  non-accredited hospitals. | CMOc1 | - | The quality of hospital care improved over time throughout the study period. The improved trend decreased post-accreditation (it stagnated). Especially, improvements were found in the quality of hospital care where the baseline performance was below best practice targets. Once the on-site survey was finished, improvements continued, but the rate levelled off. | Moderate | 13 | C1 |
| Thornlow, 2009 (USA) | Desk research | Accreditation | To examine the relationships between patient safety practices and patient safety outcomes. | CMOc1 | - | Larger hospitals demonstrated higher rates of adverse events than smaller hospitals on infections. Certain adverse events may be reduced by preventive protocols that are reflected in accreditation standards. | Low | 8 | D1 |
| Braithwaite, 2010 (Australia) | Multiple case study | Accreditation | To determine whether accreditation performance is associated with self-reported clinical performance and independent ratings of organisational performance. | CMOc1 | - | Accreditation performance was significantly positively correlated with organisational culture and leadership. There was a positive trend between accreditation and clinical performance. Accreditation was unrelated to organisational climate and consumer involvement. | Low | 9 | C1 |
| Owen, 2011 (Australia) | Descriptive study | Peer review | To compare the uptake of peer review among interns in mandatory and voluntary peer-review programmes. | CMOc7 | Physicians | 8/60 interns of the mandatory approach completed all steps of the peer review process, whereas 0/45 interns of the voluntary approach completed the process. Resistance to the peer review process occurred at all stages of the trial. However, at the voluntary site, almost all respondents ranked peer review positively, however they suggested that peer review was good for 'others' who might be struggling in certain areas of professional competency. | High | 10 | D1 |
| Gunningberg, 2008 (Sweden) | Cohort study | Clinical audit | To compare the prevalence of pressure ulcers and prevention before and after a QI programme. | CMOc2 | Physicians and nurses | The use of pressure-reducing mattresses increased after the QI programme, planned repositioning decreased. Patients who developed pressure ulcers were older, at risk for ulcers, incontinent and had a longer length of stay. Little prevention was documented at admission. | Low | 12 | C3 |
| Canitano, 2015 (Italy) | Descriptive study | Accreditation | To describe three improvement actions as recommended by the accreditation process. | CMOc1 | Physicians and nurses | An improvement plan with 26 actions was formulated. Many procedures were standardized. | Low | 5 | D2 |
| Mazzini, 2015 (Italy) | Descriptive study | Accreditation | To describe the OECI accreditation process. | CMOc5 | Physicians and nurses | Availability and willingness for exchange of views, the major investments made in recent years in the field of care and research, the range of services dedicated to the care of cancer patients were highlighted as the salient features. Among the opportunities noted, the need for investing more in care pathways, in the role of nurses, in the role of patients, and in the computerization of medical documentation were highlighted. | Low | 7 | D2 |
| Stephenson, 2016 (Australia) | Multiple case study | Clinical audit | To audit and identify barriers to compliance with best practice and to implement and assess the effects of strategies to promote best practice in falls prevention. | CMOc3 | Physicians and nurses | The mean hospital-wide fall rates did not vary between the pre-project and post-implementation periods. Although not reflected in the data, many of the clinical leaders indicated during a focus group session that falls, especially falls resulting in injury, appeared to have decreased in participating wards. | Moderate | 15 | C1 |
| Greenfield, 2015 (Australia) | Multi method study | Accreditation | To investigate the development and implementation  of an accreditation  scheme and standards, their expected benefits, and challenges  and facilitators to implementation. | CMOc1 | - | The accreditation scheme promoted greater regulatory accountability and may potentially increase the involvement of professionals in QI and provide a nationally consistent framework specifying safety and quality measures and requirements. | Low | 6 | D1 |
| Lanteigne, 2016  (Italy, Caribbean) | Multiple case study | Accreditation | To assess whether accreditation brings about change and organisational learning. | CMOc1  CMOc5 | Not described | Both organizations improved their level of compliance with standards. Individuals developed better communication skills and a better understanding of the challenges facing their institution. For the accreditation teams, group work and interdisciplinarity stood out as the strongest elements of transformation common to both organizations. | High | 9 | C1 |
| Nicolaisen, 2018 (Denmark) | Cross-sectional study | Accreditation | To examine and compare middle and senior hospital managers’ perceptions  of the effects of a mandatory accreditation programme. | CMOc2 | - | Participants perceived the accreditation as having: led to an increased focus on registration, documentation and additional and unnecessary procedures. While the accreditation was perceived as increasing a focus on quality, the time required for accreditation was at the expense of patient care. | Low | 4 | D1 |
| Dunne, 2018 (UK) | Descriptive study | Clinical audit | To assess rates of audit activity and completion and explore the barriers to successful audit completion. | CMOc2 | Physicians | Low completion of clinical audit cycle. The commonest reason cited was that the prime audit driver was a junior doctor and that these junior doctors either completed their objective or moved hospital trusts leading to a lack of audit momentum (three audits). | Low | 5 | D1 |
| Hanskamp, 2018 (Part 2) (The Netherlands) | Multiple case study | Accreditation | To identify factors that explain the observed effects of internal auditing on improving  patient safety. | CMOc2 | - | Respondents had positive audit experiences, with the exception of the amount of preparatory work by departments. Factors that hindered implementation were time-consuming and labour-intensive implementation of improvement actions; and limited organizational support for quality improvement. | Low | 12 | C1 |
| Hanskamp, 2018 (Part 1) (The Netherlands) | Multiple case study | Accreditation | To evaluate the effectiveness of internal auditing in hospital care focussed on improving  patient safety. | CMOc1 | - | Adverse events and preventable adverse events rate decreased; however, the differences before and after auditing were not statistically significant. The patient-reported experience measures regarding patient safety improved. Patient safety culture and team climate remained unchanged after the internal audit. Medication safety and information security improved. | Low | 11 | C1 |
| Stewart, 2018 (UK) | Single case study | Clinical audit | To improve the identiﬁcation and management of delirium. | CMOc2  CMOc4 | Physicians, nurses, others | The tool was deemed to be used appropriately, meaning that 60% of patients received timely assessment; 18% of patients were identiﬁed as delirious in audit one. Five months later, only 95 assessment points out of a possible 199 were being appropriately assessed (47%); however, a greater number (32%) were identiﬁed as delirious. | Moderate | 14 | C3 |
| Desveaux, 2017 (Canada) | Qualitative study | Accreditation | To explore how organisations respond to and interact with the accreditation process  and the actual and potential mechanisms through which accreditation may influence quality. | CMOc1  CMOc2  CMOc3 | - | The accreditation process is largely viewed as a quality assurance process. Coherence is established when an organization and its staff perceive that accreditation aligns with the organization’s beliefs, context and model of service delivery. Quality improvement action occurs when organizations take purposeful action in response to observations, feedback or self-reﬂection resulting from the accreditation process. | High | 8 | D1 |
| Sinuff, 2015 (Canada) | Qualitative study | Clinical audit | To understand both the experiences of ICU clinicians and leaders with audit and feedback and the core mechanisms that lead to the success or failure of the implementation of audit and feedback. | CMOc2 | Physicians and nurses | Clinicians interviewed experienced audit and feedback as fragmented and variable in its effectiveness. The audit process was perceived as being insufficiently transparent. Feedback was often untimely, incomplete and not actionable. Suggestions for improvement included improving information sharing about the rationale for change and the audit process, tools and metrics; implementing peer-to-peer quality discussions to avoid a top-down approach; providing effective feedback which contains specific, transparent and actionable information; delivering timely feedback and increasing engagement by senior management. | Moderate | 4 | D1 |
| Looper, 2016 (USA) | Descripive study | Clinical audit | To examine the administration of chemotherapy and to assess the nursing responsibilities related to this process. | CMOc2  CMOc3 | Physicians, nurses, others | Follow-up data have demonstrated that the project was highly successful and improved accuracy, patient and nurse safety, and effectiveness of chemotherapy administration. | Moderate | 13 | D2 |
| Aldridge, 2018 (Australia) | Single case study | Clinical audit | To reduce the number of paediatric respiratory viral swabs. | CMOc2 | Physicians and nurses | The use of swabs decreased. | Low | 12 | C3 |
| Chua, 2018 (USA) | Interrupted time series study | Clinical audit | To evaluate the effectiveness and feasibility of a physician-targeted quality improvement intervention with education and feedback on the prescription of beta-blockers and ACEI/ARB. | CMOc2 | Physicians | Structured education program was associated with a significant rise in beta-blockers prescription rates from a baseline, but a no sustained rise in ACEI/ARB prescription. Regular performance feedback resulted in a further sustained increase in ACEI/ ARB prescription rates. There was a reduction in 180-day readmission rates that correlated with the improvements in beta-blocker and ACEI/ARB prescription. | Low | 12 | C3 |
| Myers, 2018 (USA) | Single case study | Clinical audit | To increase venous thromboembolism prophylaxis among patients on wards. | CMOc7 | Physicians, nurses, others | Pharmacist-of-resident education elicited the largest improvement and was sustained through a recurring pharmacist-led, interprofessional educational session as part of the monthly hospital orientation for incoming residents. Data analysis showed a statistically significant increase in prophylaxis use. | Low | 14 | C3 |
| Shadman, 2016 (USA) | Single case study | Clinical audit | To increase adherence to safe sleep practices for infants. | CMOc3 | Physicians, nurses, others | Nursing knowledge of sleep practices increased significantly for each item. Significant improvements were noted in individual practices. Improvements in caregiver home practices after discharge were not statistically significant. | Low | 8 | C3 |
| Leung, 2015 (Canada) | Quasi-experimental time-series design | Clinical audit | To decrease the number of diagnostic hysteroscopies performed in the Operating Room by 75% over one year. | CMOc2 | Physicians | During the intervention period, 33 operations were performed, a 70% reduction from baseline. In the ﬁnal quarter of the intervention period, there was an 81% reduction in the number of operations with adequate preoperative evaluation compared with baseline. | Low | 15 | C3 |
| Kilsdonk, 2016 (The Netherlands) | Qualitative study | Peer review | To explore the value, perceived impact, and (future) role of  external peer review in cancer care. | CMOc2  CMOc5 | - | Improving clinical care and organization were the main motives for participation. Positive impact was perceived on multiple aspects of care. Criticism was raised on the content of the program being too theoretical and organization-focussed. According to most stakeholders, external peer review can improve multidisciplinary team work in cancer care; however, the acceptance is threatened by a perceived disbalance between effort and visible clinical impact. | Moderate | 9 | D1 |
| Counihan, 2016 (USA) | Single case study | Clinical audit | To characterize the process of surgical multidisciplinary rounds (SMDR) and to evaluate the overall effect on improving the quality of care. | CMOc2 |  | Reductions in length of stay and complications Additionally, SMDR increased awareness of Accreditation Council for Graduate Medical Education core competencies among surgical residents and was associated with enhanced job satisfaction among participants. | Low | 8 | C3 |
| Dafoe, 2015 (Australia) | Single case study | Clinical audit | To identify and implement strategies that might increase mobilisation. | CMOc3 | Physicians, nurses, others | Many mobility outcomes did not significantly change between the baseline and follow-up audits. | Low | 10 | C3 |
| Wood, 2015 (UK) | Single case study | Clinical audit | Not described | CMOc3 | Physicians, nurses, others | Since introduction of the service improvement team, consistent signs of improvement have been visible across the admission areas in four out of ﬁve of the clinical care targets. | Low | 11 | C3 |
| Wooller, 2018 (Canada) | Pre- and postintervention study | Clinical audit | to describe the use of the SafetyLEAP program to drive improvement efforts, and specifically to reduce the prevalence of urinary catheters at a large academic health center.. | CMOc2  CMOc3 | Physicians and nurses | Overall, the adherence to the SafetyLEAP program was 97.4% on both general internal medicine wards. The daily catheter point prevalence decreased from 22 to 13%. After the implementation of the program, the urinary catheter utilization ratio (defined as urinary catheter days/patient days) declined from 0.14 to 0.12. Catheter-associated urinary tract infections (CAUTI) were unchanged. | Moderate | 14 | C3 |
| Bogh, 2018 (Denmark) | Interview study | Accreditation | To understand how staff at various levels perceive and understand  hospital accreditation generally and in relation to quality improvement (QI) specifically. | CMOc2 | Physicians, nurses and other healthcare professionals | Staff reported that The Danish Healthcare Quality Programme affected management priorities: office time and working on documentation, which reduced time with patients and on improvement activities. Organisational structures were improved during preparation for accreditation. Staff perceived that the hospital was better prepared for new QI initiatives after accreditation; staff found disease specific requirements unnecessary. Other areas benefited from accreditation. Interviewees expected that organisational changes, owing to accreditation, would be sustained and that the QI focus would continue. | High | 11 | D2 |
| Smiddy, 2019 (Ireland) | Retrospective interrupted time series design | Clinical audit | To assess the relationship between an individualised OHHA and feedback intervention with doctors observed HH compliance. | CMOc2 | Physicians | HH compliance increased significantly among both orthopaedic surgeons and other consultant doctors, P < 0.05. | Low | 13 | C3 |
| Weske, 2018 (The Netherlands) | Case study | Accreditation | To contribute to the knowledge on how to increase compliance with  obligatory rules and regulations. | CMOc1 | Other healthcare professionals | Although all auditors use catalytic enforcement actions, these do not lead to (intended) compliance of all ward leaders. Rather, the catalytic actions contribute to (intended) compliance of ward leaders that are motivated, whereas they do not for the ward leaders that are not motivated. For the motivated ward leaders, catalytic enforcement actions contribute to (intended) compliance by increasing ward leaders’ knowledge of the rules and how to comply with them. | Moderate | 8 | C4 |
| Healy, 2019 (Ireland) | Case study | Clinical audit | To establish the incidence of IPH in the department and to examine how staff managed patient care to prevent IPH from occurring. | CMOc4 | Nurses | Presentation of the results of this audit has resulted in an increase in staff awareness of the incidence of IPH and an improved awareness of the patient care interventions required to prevent it. | Low | 6 | C4 |
| Conaty, 2018 (Ireland) | Case study | Clinical audit | To improve surgical antimicrobial prophylaxis (SAP) prescribing in  orthopaedic surgery using the model for improvement framework. | CMOc3 | Physicians | SAP in 168 orthopaedic surgeries from 15 June 2016 to 31 January 2017 was studied. Prescribing appropriateness improved from 20 to 78 per cent. Junior doctor changeover necessitated additional education and reminders. | Low | 13 | C3 |
| Gude, 2019 (The Netherlands) | Multiple case study | Clinical audit | To understand the mechanisms through which A&F with action implementation toolbox facilitates action planning by ICUs to increase A&F effectiveness. | CMOc3 | Physicians | ICUs with toolbox planned more actions directly aimed at improving practice (p = 0.037) and targeted a wider range of practice determinants compared to ICUs without toolbox. ICUs with toolbox also completed more actions during the study period, but not significantly (p = 0.142). ICUs without toolbox reported more difficulties in identifying what actions they could take. Regardless of the toolbox, all ICUs still experienced barriers relating to the feedback (low controllability, accuracy) and organisational context (competing priorities, resources, cost). | High | 16 | C1 |
| Dixon-Woods, 2019 (UK) | Etnographic study | Clinical audit | To explore how improvements in both data submission and completion rates were achieved during a crucial period of the evolution of two large-scale data exercises. | CMOc3 | Other healthcare professionals | Critical to the improvements in submission and completion rates in the two exercises were the efforts of clinical leaders to refigure “data work” as a professionalization strategy. Using a series of strategic manoeuvres, leaders constructed a cultural account that tied the fortunes of the healthcare professions to the submission of high-quality data, proposing that it would demonstrate responsibility, transparency, and alignment with the public interest. In so doing, clinical leadership deployed tactics that might have been seen as unwarranted managerial aggression had they been imposed by parties external to the profession. Many residual challenges were linked not to principled objection by clinicians, but to mundane problems and frustrations in obtaining, recording, and submitting  data. The cultural framing of data work as a professional duty was important to resolving its status as an abject form of labour. | Moderate | 7 | D1 |
| Tiscar-Gonzalez, 2019 (Spain) | Case study | Clinical audit | To implement evidence-based recommendations for post-surgical pain management and improve quality of care for patients. | CMOc3 | Nurses | The baseline audit revealed the need to increase health education for patients and, or, their families on managing postoperative pain, with a special emphasis on non-pharmacological measures. This education was not recorded in the patients’ medical histories at baseline and the aim was to rectify this. In follow-up audits, 100% of patients received individually tailored education about postoperative pain and its management. | Moderate | 13 | C3 |
| Ellis, 2020 (Denmark) | Multiple case study | Accreditation | To examine managers’ attitudes towards and use of a mandatory accreditation program in Denmark, the Danish Healthcare Quality Program (Den Danske Kvalitetsmodel [DDKM]) after it was terminated in 2015. | CMOc1 | Other healthcare professionals | Overall, managers’ perceptions of accreditation were favorable, highlighting key findings about some of the strengths of accreditation. DDKM was found most useful for standardizing processes, improving patient safety, and clarifying responsibility in the organization. Managers were most negative about DDKM’s ability to improve their hospitals’ financial performance, reshape the work environment, and support the function of clinical teams. Results were generally consistent across age and management level; however, managers with greater years of experience in their position had more favorable attitudes, and there was some variation in attitudes towards and use of DDKM between regions. | Low | 9 | C2 |
| Albornos-Munoz, 2018 (Spain) | Case study | Clinical audit | To improve fall prevention and management through clinical audits and the implementation of a quality-improvement cycle at the local level. | CMOc3 | Nurses | Compliance rates for the evidence-based criteria were low in the baseline audit. Five barriers were identified in relation to fall assessment and management and, based on getting research into practice, strategies were designed, developed and implemented to overcome these barriers. After implementation, most of the fall-riskassessment criteria showed an overall improvement, but there was no effect on care plan recording. Awareness of the assessment and management of fall risks were increased among professionals and patients on all three study wards. | Moderate | 14 | C3 |
| Smith, 2018 (Australia) | Case study | Clinical audit | To improve the effectiveness of nutritional screening and assessment practices through clinical audits and the implementation of evidence-based practice recommendations. | CMOc2 | Nurses | The baseline audit revealed deficits between current practice and best practice across the 10 criteria. Barriers for implementation of nutritional screening and assessment best practice criteria were identified by the focus group and an education strategy was implemented. There were improved outcomes across all best practice criteria in the follow-up audit. | Low | 13 | C3 |
| Alomari, 2020 (Australia) | An Action Research (AR) three-phase quantitative study | Clinical audit | To evaluate a bundle of interventions, developed and implemented by nurses, to reduce medication administration error rates and improve nurses’ medication administration practice | CMOc3  CMOc4 | Nurses | Postimplementing the interventions, medication error rates were reduced by 56.9% despite an increase in the number of patient admissions and in the number of prescribed medications. The rate of medication errors per 1,000 prescribed medications significantly declined from 2014 to 2016. The ward nurses were more compliant with the policy in postintervention phase than preintervention phase. The improvement in SAQ was reported in five of the seven domains. | Moderate | 13 | C3 |
| Currie, 2020 (UK) | Exploratory qualitative study | Clinical audit | To explain the mechanisms influencing implementation of a national programme for AMS in acute-care hospitals across Scotland, using NPT as an interpretive framework to explore multiprofessional perspectives. | CMOc2 | Physicians nurses and other healthcare professionals | Results indicated that major barriers relate to organisational context and resource availability. AMS had coherence for implementation leads and prescribing doctors; less so for consultants and nurses who may not access training. Conflicting priorities made obtaining buy-in from some consultants difficult; limited role perceptions meant few nurses or clinical pharmacists engaged with AMS. Collective individual and team action to implement AMS could be constrained by lack of medical continuity and hierarchical relationships. Reflexive monitoring based on audit results was limited by the capacity of AMS Leads to provide direct feedback to practitioners. | High | 8 | D1 |
| Rohweder, 2019 (USA) | pre-test/post-test single group design and mixed methods data collection. | Clinical audit | To describe the strategies used by the collaborative and explore the mechanisms through which the collaborative led to improvements in colorectal cancer (CRC) screening rates. | CMOc2 | Unknown | Teams completed all four QIC tools: aim statements, process maps, gap and root cause analysis, and Plan-Do-Study-Act cycles. FQHCs increased their uptake of evidence-based CRC screening interventions and rates increased 8.0% between 2017 and 2018. Focus group findings provided insights into participants' opinions regarding the feasibility and appropriateness of the implementation strategies and how they influenced outcomes. Results support the collaborative's positive impact on FQHC capacity to implement QI tools and EBIs to improve CRC screening rates. | Low | 13 | C1 |
| Mughal, 2018 (UK) | Survey design | Clinical audit | To evaluate the use of a weekend handover proforma in General Surgical patients at a University Teaching Hospital. | CMOc2 | Physicians | There was 70% compliance with the new standardized proforma with a median handover score of 83% (IQR = 0‐100). The results were presented at a clinical governance meeting, and the proforma was refined. After this change, the proforma was used in 71% of patients, and the median score was 65% (IQR = 0‐80, P = 0.0516). Compliance after an email reminder was 69%, and median handover score was 80% (IQR = 0‐90, P = 0.1037). After induction training, there was a significant improvement in proforma compliance (94%) and median score (90%, IQR = 80‐90, P = 0.013). | Low | 13 | C3 |
| Gazarin, 2020 (Canada) | Case study | Clinical audit | To increase the pre‑operative checklist completion in its entirety to 80% by April 2017 and to provide a detailed description of our results throughout this 12‑month QI study. | CMOc7 | Physicians and nurses | Remarkably, completion of the pre‑operative checklist increased from 25% to 67% and finally to 94%. Furthermore, the previous chart’s presence and completion of pre‑operative orders improved from 87% to 100% and from 82% to 99%, respectively. Another significantly important secondary outcome was improvement in interdepartmental relationships and collaboration. With better communication and checklist completion rates, there came increased patient preparedness and improved efficiency. | High | 9 | C3 |

Abbreviations: No., Number. QI., Quality Improvement.

^a^ Quality Improvement Minimum Quality Criteria Set (QI-MQCS) score (possible range 0–16) (Hempel et al. (2015))

^b^ The level of empirical evidence based on criteria established by the Cochrane ‘Effective Practice and Organisation of Care’ (EPOC) review group (EPOC (2013))
