# Supplementary material for: Understanding how and why audits work in improving the quality of hospital care: A systematic realist review
Source: PLoS One. 2021 Mar 31;16(3):e0248677. doi: 10.1371/journal.pone.0248677 (PMC8011742; doi:10.1371/journal.pone.0248677)
Supplement: S2 Table — (DOCX) [file pone.0248677.s004.docx]

**S2 Table. List of codes**

Some of the codes do have overlap because of the different terms for describing aspects of the audit used in our included articles. Therefore, the categories are not mutually exclusive categories but are complementary.

| Codenumber | Code | Frequency |
| --- | --- | --- |
| C1 | Context - Bottom-up initiative | 7 |
| C2 | Context - Commitment before participate | 1 |
| C3 | Context - Deficiencies in quality of care | 26 |
| C4 | Context - Enough resources | 1 |
| C5 | Context - Experience with audit methodology | 12 |
| C6 | Context - External pressure/Top down initiative | 18 |
| C7 | Context - Lack of awareness | 1 |
| C8 | Context - Lack of education/knowledge | 6 |
| C9 | Context - Lack of knowledge regarding audit methodology | 2 |
| C10 | Context - Lack of resources | 12 |
| C11 | Context - Leadership not involved in QI | 5 |
| C12 | Context - Motivation to participation: Joined responsibility | 1 |
| C13 | Context - National awareness for indicators | 9 |
| C14 | Context - No enthusiastic healthcare professionals | 2 |
| C15 | Context - No experience with audit methodology | 5 |
| C16 | Context - No QI culture | 2 |
| C17 | Context - No safe learning culture | 3 |
| C18 | Context - Open culture to change/ safe learning culture | 14 |
| C19 | Context - Poor adherence of patients | 1 |
| C20 | Context - Small organisation > easy to know each other | 1 |
| C21 | Context - Stability among staff | 1 |
| C22 | Context - Staff feel responsible for local population | 1 |
| C23 | Context - Strong leadership for QI | 26 |
| C24 | Context - Well delineated guidelines | 2 |
|  |  |  |
| Res1 | Resource - Addressing punitive culture | 6 |
| Res2 | Resource – Anonymity | 8 |
| Res3 | Resource - Audit leader: Interested staff members | 9 |
| Res4 | Resource - Audit subject recognised issue | 5 |
| Res5 | Resource - Audit subject regularly emphasised | 2 |
| Res6 | Resource - Clear expectations of the audit | 2 |
| Res7 | Resource - Clear input from the work floor | 5 |
| Res8 | Resource - Close collaboration between audit leaders and work floor | 6 |
| Res9 | Resource - Committee of peers | 1 |
| Res10 | Resource - Conducted by healthcare professionals | 19 |
| Res11 | Resource - Consequences for healthcare professionals if recommendations weren't followed | 2 |
| Res12 | Resource - Creation of good climate | 1 |
| Res13 | Resource - Deemphasizing individual scores | 4 |
| Res14 | Resource - Different types of feedback | 1 |
| Res15 | Resource - Doctors and nurses in charge | 3 |
| Res16 | Resource - Downplaying official roles/hierarchy | 5 |
| Res17 | Resource - Education about audit subject | 32 |
| Res18 | Resource - Education in methodology | 5 |
| Res19 | Resource - Empower physicians | 1 |
| Res20 | Resource - Encouragement to attend audit meeting | 1 |
| Res21 | Resource - Encouragement to give feedback to other staff | 1 |
| Res22 | Resource - Experienced auditor/reviewer | 1 |
| Res23 | Resource - External support with audit methodology | 6 |
| Res24 | Resource - Feedback of healthcare professionals about audit process | 7 |
| Res25 | Resource - Feedback to stakeholders | 29 |
| Res26 | Resource - Financial bonus for participation | 3 |
| Res27 | Resource - Flexible approach | 3 |
| Res28 | Resource - Good skills of auditor | 1 |
| Res29 | Resource - Individual data is examined | 6 |
| Res30 | Resource - Information sessions | 3 |
| Res31 | Resource - Knowledge networking | 9 |
| Res32 | Resource - Management support | 2 |
| Res33 | Resource - Mandatory audit | 5 |
| Res34 | Resource - Medical support | 2 |
| Res35 | Resource – Multidisciplinary audit leads | 11 |
| Res36 | Resource - No encouragement from senior staff | 1 |
| Res 37 | Resource - No face-to-face feedback | 1 |
| Res38 | Resource - No individual feedback | 4 |
| Res39 | Resource - Nominated champions | 18 |
| Res40 | Resource - Nurse managers in charge | 3 |
| Res41 | Resource - Ownership of changes by healthcare professionals | 6 |
| Res42 | Resource - Patient education | 3 |
| Res43 | Resource - Peers to assess performance | 2 |
| Res44 | Resource - Peer-to-peer discussion | 2 |
| Res45 | Resource - Research department in charge | 1 |
| Res46 | Resource - Safety specialist | 1 |
| Res47 | Resource - Selection of motivated leaders | 1 |
| Res48 | Resource - Senior staff encouraged audit | 5 |
| Res49 | Resource - Standardisation of documentation | 5 |
| Res50 | Resource - Strategies were used in to ensure that audit results were accepted by healthcare professionals | 12 |
| Res51 | Resource - Structural attention for safety | 1 |
| Res52 | Resource - Trained auditors | 2 |
| Res53 | Resource - Transparent audit process | 2 |
| Res54 | Resource - Usage of a previous developed audit format | 1 |
| Res55 | Resource - Voluntary audit | 1 |
| Res56 | Resource - Working group with people less close to work floor | 5 |
| Res57 | Resource -External support audit methodology | 3 |
|  |  |  |
| Reas1 | Reasoning - Audit is additional burden | 4 |
| Reas2 | Reasoning - Audit is exercise, no driver of change | 1 |
| Reas3 | Reasoning - Audit teams mobilised colleagues | 2 |
| Reas4 | Reasoning - Departmental drive to QI | 3 |
| Reas5 | Reasoning - Disinterest | 1 |
| Reas6 | Reasoning - Driving force junior doctor | 2 |
| Reas7 | Reasoning - Engagement | 19 |
| Reas8 | Reasoning - Enthusiasm suggested improvements | 1 |
| Reas9 | Reasoning - Good for others | 1 |
| Reas10 | Reasoning - Good participation rate | 5 |
| Reas11 | Reasoning – Hostility | 1 |
| Reas12 | Reasoning - Importance for patient care | 3 |
| Reas13 | Reasoning - Lack of credibility | 1 |
| Reas14 | Reasoning - Lack of transparency | 3 |
| Reas15 | Reasoning - Learn from colleagues | 13 |
| Reas16 | Reasoning - Link accreditation to changes made | 2 |
| Reas17 | Reasoning - Morale booster | 2 |
| Reas18 | Reasoning - Mutual understanding of problems | 1 |
| Reas19 | Reasoning - Not always clinically relevant | 7 |
| Reas20 | Reasoning - Not engaged healthcare professionals | 4 |
| Reas21 | Reasoning - Reluctance to participate | 1 |
| Reas22 | Reasoning - Rewarding | 2 |
| Reas23 | Reasoning - Strong ties between interns | 1 |
| Reas24 | Reasoning - Threat to career prospects | 1 |
| Reas25 | Reasoning - Trust | 3 |
| Reas26 | Reasoning - Increased workload | 7 |
|  |  |  |
| Prox1 | Proximal outcome - Audit and (ongoing) feedback common practice | 3 |
| Prox2 | Proximal outcome - Audit objectives achieved within time | 4 |
| Prox3 | Proximal outcome - Audit was too time consuming | 3 |
| Prox4 | Proximal outcome - Better understanding of organisational challenges | 3 |
| Prox5 | Proximal outcome - Cultural change | 1 |
| Prox6 | Proximal outcome - Establishment QI committee | 3 |
| Prox7 | Proximal outcome - Few interactions between doctors and nurses | 2 |
| Prox8 | Proximal outcome - Group work and interdisciplinarity | 13 |
| Prox9 | Proximal outcome - Improved reporting | 6 |
| Prox10 | Proximal outcome - Improved utilisation | 1 |
| Prox11 | Proximal outcome - Increase unnecessary procedures | 1 |
| Prox12 | Proximal outcome - Increased awareness | 13 |
| Prox13 | Proximal outcome - Increased commitment to quality | 4 |
| Prox14 | Proximal outcome - Increased critical thinking | 3 |
| Prox15 | Proximal outcome - More active pursuit to QI | 9 |
| Prox16 | Proximal outcome - More active pursuit to the audit subject | 1 |
| Prox17 | Proximal outcome - Poor participation | 4 |
| Prox18 | Proximal outcome - Priorisation of care | 1 |
| Prox19 | Proximal outcome - Reduced fear of retribution | 1 |
| Prox20 | Proximal outcome - Skills development | 4 |
| Prox21 | Proximal outcome - Speed of implementation | 4 |
| Prox22 | Proximal outcome - Stronger position of staff | 2 |
| Prox23 | Proximal outcome - Translation of audit results into improvement actions | 1 |
| Prox24 | Proximal outcome - Waiting for leaders to adopt improvements | 1 |
|  |  |  |
| O1 | Outcome - Audit cycle not complete | 2 |
| O2 | Outcome - Better work hours | 1 |
| O3 | Outcome - Compliance (sustained after the initial project) | 9 |
| O4 | Outcome - Encouragement of leadership | 2 |
| O5 | Outcome - Improved compliance | 23 |
| O6 | Outcome - Improved medication safety | 1 |
| O7 | Outcome - Less medical errors | 2 |
| O8 | Outcome - Low uptake and completion audit | 3 |
| O9 | Outcome - Management level | 1 |
| O10 | Outcome - More resources available | 7 |
| O11 | Outcome - No change in patient safety culture | 1 |
| O12 | Outcome - No change in team climate | 1 |
| O13 | Outcome - No improved reporting | 1 |
| O14 | Outcome - No improved treatment availability | 1 |
| O15 | Outcome - No support for other QI initiatives | 2 |
| O16 | Outcome - Reduction in costs | 1 |
| O17 | Outcome - Spread after the initial project | 4 |
|  |  |  |
| Add1 | Additional info - Audit goal not clear | 3 |
| Add2 | Additional info – Audit helped to accelerate improvements | 1 |
| Add3 | Additional info - Audit subject multidisciplinary team responsibility | 3 |
| Add4 | Additional info - Collaboration is essential | 5 |
| Add5 | Additional info - Flexible approach is important for fit | 4 |
| Add6 | Additional info - Gut feelings supported by data | 3 |
| Add7 | Additional info - Less sense of urgency after the audit is finished | 1 |
| Add8 | Additional info - Openly modelled and driven by senior staff | 7 |
| Add9 | Additional info - Ownership | 1 |
| Add10 | Additional info - Peer review overestimation of compliance | 1 |
| Add11 | Additional info - Shared vision not enough, knowledge is needed | 1 |
| Add12 | Additional info - Suggestions: champion/staff is important | 8 |
| Add13 | Additional info - System redesign most important factor in success | 1 |
| Add14 | Additional info - Different tasks middle and senior managers | 1 |
